# Thermal enhancement in Falkner–Skan flow of the nanofluid by considering molecular diameter and freezing temperature

**DOI:** 10.1038/s41598-022-13423-7

**Published:** 2022-06-08

**Authors:** Adnan, Rashid Murtaza, Iftikhar Hussain, Ziaur Rehman, Ilyas Khan, Mulugeta Andualem

**Affiliations:** 1 Department of Mathematics, Mohi-ud-Din Islamic University, Nerian Sharif, AJ&K 12080 Pakistan; 2 Department of Civil and Environmental Engineering, College of Engineering, Majmaah University, Majmaah, Al-Majmaah, 11952 Saudi Arabia; 3 Department of Mathematics, College of Science Al-Zulfi, Majmaah University, Al-Majmaah, 11952 Saudi Arabia; 4 Department of Mathematics, Bonga University, Bonga, Ethiopia

**Keywords:** Engineering, Mathematics and computing, Nanoscience and technology

## Abstract

The analysis of nanofluids heat transfer over a wedge is very important due to their wider applications in applied thermal engineering, chemical engineering and biomedical engineering etc. Therefore, aim of the study is to explore the heat transport in nanofluid over a wedge (Falkner Skan flow) under viscous dissipation and thermal radiation over a wedge. The proper model formulation is carried out via similarity relations and empirical correlations of the nanofluids. After successful model transformation, numerical scheme (RK technique along with shooting technique) applied and furnished the results over the desired domain under varying effects of preemenant flow parameters. The results revealed that the velocity rises for opposing ($\gamma <0$) and assisting ($\gamma >0$) flows against $\lambda$ and significant contribution of Ec and imposed thermal radiations (Rd number) observed in thermal performance of the nanofluid. The temperature declines by strengthen $\lambda$ and optimum decrement is noted for opposing flow. Finally, a comparison is provided for various values of $\lambda$ ($\lambda =\mathrm{0,0.014}, 0.04, 0.09, 0.1429, 0.2, \mathrm{0.333,0.5}$) with previously published work under certain restrictions and found an excellent agreement.

## Introduction

Due to wide application of nanofluids in a series of industrial and technological processes, the research of nanofluids is of great significance and cannot be ignored. Nanofluids are defined as fluids containing nano-sized particles, called nanoparticles. The nanofluid produces a colloidal suspension of tiny particles in the regular liquid. Water, ethylene glycol etc. are typical choices for base fluids. The analysis of magneto hydrodynamic Falkner Skan is one of the major and basic motives due to its uses in various industries and practical situation. Especially, traditional flow of Non-Newtonian and Newtonian fluids over a wedge presently attains fame among the researches. The forerunner work in this period was done in^1^. Later on, it was improved by Rajagopal^2^. They investigated the dynamics of non-Newtonian liquid flowing over a wedge.

The researchers concentrated on the dynamics of liquids under certain flow conditions. The similarity solutions for wedge flow modeled by improving the strength of Pr examined in^3^. They developed the particular model by taking convective heat transport and higher Pr values. The behaviour of nanofluid characteristics under the impacts of Lorentz forces discussed in^4^. They modeled the problem over a wedge under free convection scenario. They furnished the results by altering the governing quantities and proved a detailed discussion. The study of wall stresses and temperature behaviour in incompressible fluid over a permeable wedge is reported in^5^. El-Dabe et al.^6^ explored the analysis of boundary layer flow of non-newtonian fluid and found hidden impacts of Lorentz forces for thermal and mass transportation. They conducted the numerical analysis of the model and then compared the outcomes with some existing relevant literature. The characteristics of casson liquid due to symmetric wedge are discussed in^7^. They concluded that the temperature of the fluid elevated due to higher prandtl effects and the walls shear stresses improved by strengthening the casson parameter.

The significant investigation of thermal transportation under combined convection and MHD over a permeable stretchable wedge is explored by Su et al.^8^. To improve thermal performance of the fluid, they plugged the influences of thermal radiations and resistive heating the constitutive correlations and then performed mathematical study and decorated the pictorial results against the pertinent governing quantities. The alterations in the fluid behaviour due to non-stationary wedge are detected in^9^. They developed the model for micropolar liquid under certain physical scenario and then discussed the dynamics of the model via graphs. Porosity of the surface imperatively alters the fluid behaviour. Therefore, Rashidi et al.^10^ organized the analysis of viscoelastic liquid over a porous wedge. In addition, they emerged the effects of thermal radiations in the model and examined the fascinating results for the fluid behaviour over the desired region. Some imperative investigations of the liquid flowing over or between the geometries by contemplating various flow conditions are presented in^11–17^ and the studies reported they’re in and tackled by implementing various mathematical techniques.

Thermal enhancement in the nanoliquid saturated by aluminum alloys is presented in^18^. They established the model under various conditions and pictorially discussed the dynamics of the liquid. In 2017, the analysis of Ferro fluid is reported by considering the properties of Lorentz forces and thermal radiation^19,20^. Recently, in 2017, Khan et al.^21^ described the study of three-dimensional squeezed in the existence of γ-Aluminum as a nanoparticle and used water, ethylene and glycol as base fluids.

Interaction of ferromagnetic nanomaterial with species under the action of chemical species report by Tahir et al.^22^. The problem developed over a stretchable cylinder and discussed the dynamics of the fluids for various values of the permanent flow quantities. Cattaneo Christov heat flux model is a potential area of research and imperatively changes the behaviour of fluid temperature. Therefore, the study of thermal transport in micropolar fluid by inducing CC model is examined by Ahmad et al.^23^ in 2021. Numerical treatment of a mathematical model for heat transport in a square duct is conducted by Fuzhang et al.^24^. Some significant recent studies regarding micropolar fluid under temperature dependent characteristics, Carbon nanotubes under bi-stratification and FVM examined in^25–27^. The investigation of thermal radiations and their contribution in the heat transfer attained much interest of the researcher community in recent time. Therefore, Acharya et al.^28^ reported the temperature behaviour under in radiated nanofluid by using thermal conductance model comprising the influences of nanolayer and diameter. Other recent studies for heat transfer under solid–liquid interfacial layer, solar energy and ferro fluid flow slippery geometry were described in^29–31^.

From the investigation of the above cited literature, the following research gap and research questions are found that will be addressed in this study:

- The heat transport by inducing thermal conductivity model (including Brownian motion, freezing temperature and molecular diameter) under novel effects of thermal radiation and viscous dissipation for opposing and assisting flow is reported over a wedge so far.
- What will be the behaviour of nanofluids velocity over a wedge for assisting/opposing and stationary cases?
- What will be the significant contribution of thermal radiations and viscous dissipation in the thermal performance of the nanofluids?
- Numerical technique will be adopted for mathematical treatment of the model.
- Is the study will be valid after imposing certain restriction on the model?

## Mathematical modelling

Consider the flow of water suspended by Aluminum oxide ${Al}_{2}{O}_{3}$ nanoparticles. It is assumed that the flow is viscous and incompressible, the particular nanofluid flow taken over the wedge geometry. The wedge is situated in Cartesian coordinate system. The velocity at the wedge is ${{u}^{*}}_{w}={{U}^{*}}_{w}{x}^{m}$ and the velocity of the nanofluid at free stream is denoted by ${{{{U}^{*}}_{w}=U}^{*}}_{\infty }{x}^{m}$, here ${{U}^{*}}_{w}$ and ${{U}^{*}}_{\infty }$ are invariable.

**Figure 1:**
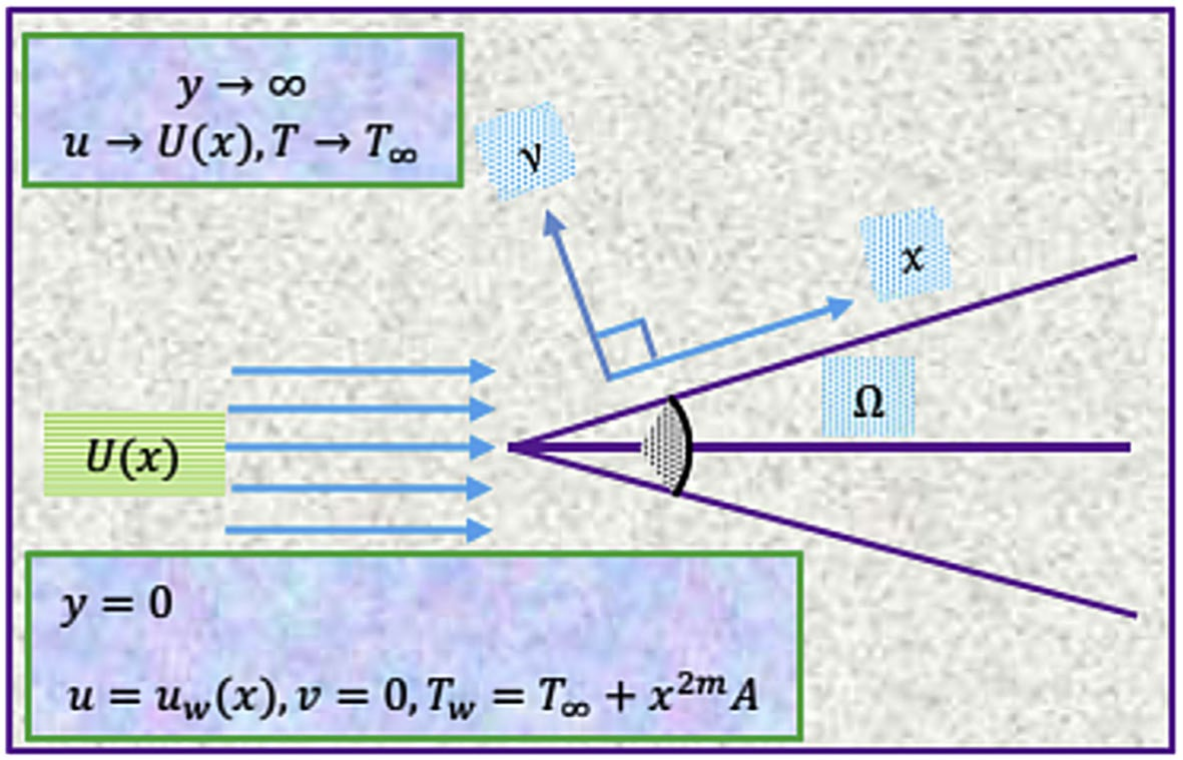
Nanofluid flow scenario.

Moreover here ${\lambda }^{*}=2m{\varnothing }^{*}$, where ${\varnothing }^{*}={(m+1)}^{-1}$ is called Hartree pressure gradient parameter corresponding to ${\lambda }^{*}=\Omega /\uppi$, where $\Omega$ is the total wedge angle. The temperature at the wedge surface is ${{\check{T}}_{w}}\left(x\right)={{\check{T}}_{\infty }}+A{x}^{2m}$ in which free surface temperature is ${{\check{T}}_{\infty }}.$ Physical configuration of the flow is pictured in Fig. 1.

In the light of above-mentioned assumptions, PDE’s representing the flow nanofluid in the presence of Hartree pressure parameter in the momentum equation and the radiative heat flux incorporated in energy equation are as follows^32,33^:

1 $$\frac{\partial {u}^{*}}{\partial x}+\frac{\partial {v}^{*}}{\partial y}=0$$

2 $${u}^{*}\frac{\partial {u}^{*}}{\partial x}+{v}^{*}\frac{\partial {v}^{*}}{\partial y}=U\left(x\right)\frac{dU\left(x\right)}{d\left(x\right)}+\frac{{\mu }_{nf}}{{\rho }_{nf}}\left(\frac{{\partial }^{2}{u}^{*}}{\partial {y}^{2}}\right)$$

3 $${u}^{*}\frac{\partial {\check{T}}}{\partial x}+{v}^{*}\frac{\partial {\check{T}}}{\partial y}=\frac{{k}_{nf}}{{\left({\rho C}_{p}\right)}_{nf}}\left(\frac{{\partial }^{2}{\check{T}}}{\partial {y}^{2}}\right)+\frac{1}{{\left({\rho C}_{p}\right)}_{nf}}{\left(\frac{\partial {u}^{*}}{\partial y}\right)}^{2}+\frac{16{\sigma }^{*}{{\check{T}}}_{\infty }^{3}}{3k{\left({\rho C}_{p}\right)}_{nf}}\left(\frac{{\partial }^{2}{\check{T}}}{\partial {y}^{2}}\right)$$

The conditions at the wedge surface ($y=0$)

$${u}^{*}={u}_{\omega }^{*}\left(x\right),$$

$${v}^{*}=0,$$

4 $${\check{T}}={T}_{\infty }+\frac{A}{{x}^{-2m}}$$

The conditions at the free stream ($y\to \infty$):

5 $${u}^{*}\to {U}^{*}\left(x\right), {\check{T}}\to {{\check{T}}}_{\infty }$$

The following similarity variables and stream function support the flow model^32,33^:

$${u}^{*}=\frac{\partial {\psi }^{*}}{\partial y},$$

$${v}^{*}=-\frac{\partial {\psi }^{*}}{\partial x},$$

$${\psi }^{*}=\sqrt{\frac{2{v}_{f}x{U}^{*}\left(x\right)}{\left(m+1\right)}} Y\left(\zeta \right)$$

$$\eta =\sqrt{\frac{\left(m+1\right){U}^{*}\left(x\right)}{2{v}_{f}x}} y,$$

6 $$Z\left(\zeta \right) =\frac{ {\check{T}}-{{\check{T}}}_{\infty }}{ {{\check{T}}}_{\omega }-{{\check{T}}}_{\infty }}$$

Since $${u}^{*}=\frac{\partial {\psi }^{*}}{\partial y}$$

So $${u}^{*}={U}_{\infty }\sqrt{{x}^{2m+1}} .{Y}^{\bullet }(\zeta )$$

The derivative of ${u}^{*}$ w.r.t $x$

7 $$\frac{\partial { u}^{*}}{\partial x}=m{{U}_{\infty }}^{*}{x}^{m-1}{Y}^{^{\prime}}\left(\zeta \right)+{{U}_{\infty }}^{*}{x}^{m}{Y}^{{^{\prime}}{^{\prime}}}\left(\zeta \right)\sqrt{\frac{\left(m+1\right){{U}_{\infty }}^{*}}{2{\nu }_{f}}y\left(\frac{m-1}{2}\right){x}^{\frac{m-3}{2}}}$$

The derivative of ${u}^{*}$ w.r.t $y$

8 $$\frac{\partial { u}^{*}}{\partial y}={{U}_{\infty }}^{*}{x}^{m}{Y}^{{^{\prime}}{^{\prime}}}\left(\zeta \right)\sqrt{\frac{\left(m+1\right){{U}_{\infty }}^{*}}{2{\nu }_{f}}{x}^{m-1} }$$

Second derivative of ${u}^{*}$ w.r.t $y$

9 $$\frac{{\partial }^{2}{u}^{*}}{\partial {y}^{2}}={{{(U}_{\infty }}^{*})}^{2}{x}^{2m-1}\frac{\left(m+1\right)}{2{\nu }_{f}}{Y}^{{^{\prime}}{^{\prime}}{^{\prime}}}\left(\zeta \right)$$

Differentiating $${\check{T}}$$ w.r.t $$x$$

10 $$\frac{\partial {\check{T}}}{\partial x}=\frac{-2mA}{{x}^{2m+1}}$$

Derivative of $${\check{T}}$$ w.r.t $$y$$

11 $$\frac{\partial {\check{T}}}{\partial y}=0$$

Second derivative of ${\check{T}}$ w.r.t $y$

12 $$\frac{{\partial }^{2}{\check{T}}}{\partial {y}^{2}}=0$$

The following empirical correlations for nanoliquid utilized^34,35^:

13 $${\rho }_{nf}=\left(1-\varnothing \right){\rho }_{f}+{\varnothing \rho }_{s}$$

14 $$\frac{{\mu }_{nf}}{{\mu }_{f}}=\frac{1}{(1-34.87\left({\left(\frac{{d}_{p}}{{d}_{f}}\right)}^{-0.3}{\varnothing }^{1.03}\right)}$$

Thermal conductivity ratio of the particular nanofluid problem already calculated^34,35^:

15 $${\left({\rho C}_{p}\right)}_{nf}=\left(1-\varnothing \right){\left({\rho C}_{p}\right)}_{f}+\varnothing {\left({\rho C}_{p}\right)}_{s}$$

16 $$\frac{{k}_{nf}}{{k}_{f}}=1+4.4{Re}_{B}^{0.04}{P}_{r}^{0.66}{\left(\frac{{\check{T}}}{{{\check{T}}}_{fr}}\right)}^{10}\left({\frac{{k}_{p}}{{k}_{f}}}^{0.03}\right){\varnothing }^{0.66}$$

where ${Re}_{B}$ is described as:

17 $${Re}_{B}=\frac{{{\rho }_{f}{u}_{B}{d}_{p}}}{{\mu }_{f}}$$

In Eq. (3), ${u}_{B}$ represents the Brownian velocity of nanoparticles and is calculated as:

18 $${u}_{B}=\frac{2{k}_{b}{\check{T}}}{\pi {\mu }_{f}{d}_{{p}^{2}}}$$

where, ${k}_{b}=1.380648\times {10}^{-23}\left(J/K\right)$ is the Boltzmann Constant. ${l}_{f} = 0.17 nm$ is the mean path of fluid particles. ${d}_{f}$ is the molecular^34,35^ diameter of water:

19 $${d}_{f}=\frac{6M}{N\pi {\rho }_{f}}$$

The value of ${d}_{f}$ is defined as

20 $${d}_{f}={(\frac{6\times 0.01801528}{6.022\times {10}^{23}\times \pi \times 898.26})}^\frac{1}{3}$$

21 $${{d}_{f}=3.85\times 10}^{-10}m$$

By using the derivatives calculated in Eqs. (7) to (12)

22 $${Y}^{{^{\prime}}{^{\prime}}{^{\prime}}}+\frac{\left[1-\varnothing +\frac{{\varnothing \rho }_{s}}{{\rho }_{f}}\right]}{(1-34.87\left({\left(\frac{{d}_{p}}{{d}_{f}}\right)}^{-0.3}.{\varnothing }^{1.03}\right)}\left(Y{Y}^{{^{\prime}}{^{\prime}}}+\lambda \left(1-{Y}^{{^{\prime}}2}\right)\right)=0$$

23 $$\left[1+Rd{A}_{2}\right]{Z}^{{^{\prime}}{^{\prime}}}+{A}_{2}\left[\frac{\left(PrY{Z}^{^{\prime}}-2\lambda PrZ{Y}^{^{\prime}}\right)}{{\left\{\left(1-\varnothing \right)+\frac{\varnothing {\left(\rho {C}_{p}\right)}_{s}}{{\left(\rho {C}_{p}\right)}_{f}}\right\}}^{-1}}+PrEc{Z}^{{^{\prime}}{^{\prime}}2}\right]=0$$

Here,

$$and \,\,\, {A}_{2}={\left[1+4.4{Re}_{B}^{0.04}{P}_{r}^{0.66}{\left(\frac{{\check{T}}}{{{\check{T}}}_{fr}}\right)}^{10}\left({\frac{{k}_{p}}{{k}_{f}}}^{0.03}\right){\varnothing }^{0.66} \right]}^{-1}$$

Further, the conditions reduced as:

At the wedge surface;

24 $$Y\left(\zeta \right)=0, { Y}^{^{\prime}}\left(\zeta \right)=\gamma , Z\left(\zeta \right)=1, as \zeta =0$$

Far from the surface:

25 $${Y}^{^{\prime}}\left(\zeta \right)\to 1, Z\left(\zeta \right)\to 0 as \zeta \to \infty$$

where, $\gamma =\frac{{u}_{w}^{*}}{{U}_{\infty }^{*}}$ (moving wedge parameter), $Pr=\frac{{\mu }_{f}{\left({\rho c}_{p}\right)}_{f}}{{k}_{f}},$ (Prandtl number) $Ec=\frac{{{u}^{*}}^{2}}{{\left({c}_{p}\right)}_{f}({{\check{T}}}_{w}-{{\check{T}}}_{\infty })}$ (Eckert number) and $Rd=\frac{16{\sigma }^{*}{T}_{\infty }^{3}}{3{k}^{*}{k}_{f}}$ (Radiation number).

Thermophysical values of the hosting liquid and nanoparticles are given as^36^ (Table 1).

**Table 1:** Thermophysical values of the particles and hist liquid.

Host liquid/nanoparticles	Thermal expansion coefficient (K^−1^)	Thermal conductivity (Wm^−1^ K^−1^)	Heat capacity (J kg^−1^ K^−1^)	Density (kg m^−3^)	Pr
Water	$20.6\times {10}^{-5}$	0.60	4182	998.3	6.96
Al_2_O_3_	$0.85\times {10}^{-5}$	40	765	3970	–

## Mathematical investigation of the model

For mathematical investigation of the model, the following procedure is adopted:

26 $${{\check{z}}}_{1}=Y, {{\check{z}}}_{2}={Y}^{^{\prime}}, {{\check{z}}}_{3}={Y}^{{^{\prime}}{^{\prime}}}, {{\check{z}}}_{4}=Z, {{\check{z}}}_{5}={Z}^{^{\prime}}$$

and

27 $${Y}^{{^{\prime}}{^{\prime}}{^{\prime}}}=\frac{\left[1-\varnothing +\frac{{\varnothing \rho }_{s}}{{\rho }_{f}}\right]}{(1-34.87\left({\left(\frac{{d}_{p}}{{d}_{f}}\right)}^{-0.3}{\varnothing }^{1.03}\right)}\left(Y{Y}^{{^{\prime}}{^{\prime}}}+\lambda \left(1-{Y}^{{^{\prime}}2}\right)\right)$$

28 $${Z}^{{^{\prime}}{^{\prime}}}=-\left[\frac{1}{\left[1+Rd{A}_{2}\right]}\right]\left[{A}_{2}\left[\frac{\left(PrY{Z}^{^{\prime}}-2\lambda PrZ{Y}^{^{\prime}}\right)}{{\left\{\left(1-\varnothing \right)+\frac{\varnothing {\left(\rho {C}_{p}\right)}_{s}}{{\left(\rho {C}_{p}\right)}_{f}}\right\}}^{-1}}\right]+PrEc{Z}^{{^{\prime}}{^{\prime}}2}\right]$$

Finally, the following version is attained:

29 $$\left[\begin{array}{c}\begin{array}{c}\begin{array}{c}{{{\check{z}}{^{\prime}}}}_{1}\\ {{{\check{z}}{^{\prime}}}}_{2}\end{array}\\ {{{\check{z}}{^{\prime}}}}_{3}\end{array}\\ {{{\check{z}}{^{\prime}}}}_{4}\\ {{{\check{z}}{^{\prime}}}}_{5}\end{array}\right]=\left[\begin{array}{c}{{\check{z}}_{2}}\\ {{{\check{z}}}_{3}}\\ \frac{\left[1-\varnothing +\frac{{\varnothing \rho }_{s}}{{\rho }_{f}}\right]}{(1-34.87\left({\left(\frac{{d}_{p}}{{d}_{f}}\right)}^{-0.3}{\varnothing }^{1.03}\right)}\left({{{\check{z}}}}_{1}{{{\check{z}}}}_{2}-\lambda \left(1-{{{{\check{z}}}_{2}}}^{2}\right)\right)\\ {{{\check{z}}}_{5}}\\ -(\frac{1}{\left[1+Rd{A}_{2}\right]}){A}_{2}[\frac{\left(PrY{{{\check{z}}}}_{5}-2\lambda PrZ{{{\check{z}}}}_{2}\right)}{{\left\{1-\varnothing +\frac{\varnothing (\rho {{C}_{p})}_{s}}{{\left(\rho {C}_{p}\right)}_{f}}\right\}}^{-1}}+PrEc{z}_{5}^{{^{\prime}}2}]\end{array}\right]$$

With conditions:

30 $$\left[\begin{array}{c}\begin{array}{c}\begin{array}{c}{{{\check{z}}}}_{1}\\ {{{\check{z}}}}_{2}\end{array}\\ {{{\check{z}}}}_{3}\end{array}\\ {{{\check{z}}}}_{4}\\ {{{\check{z}}}}_{5}\end{array}\right]=\left[\begin{array}{c}\begin{array}{c}\begin{array}{c}0\\ \gamma \end{array}\\ {\eta }_{1}\end{array}\\ 1\\ {\eta }_{1}\end{array}\right]$$

## Graphical results with discussion

### Analysis of results

This section devoted to analyze the behaviour of the nanofluid velocity $Y{^{\prime}}(\zeta )$ and temperature field $Z(\zeta )$ against the preemenant parameters for feasible range.

### Discussion of results

#### The velocity field

**Figure 2:**
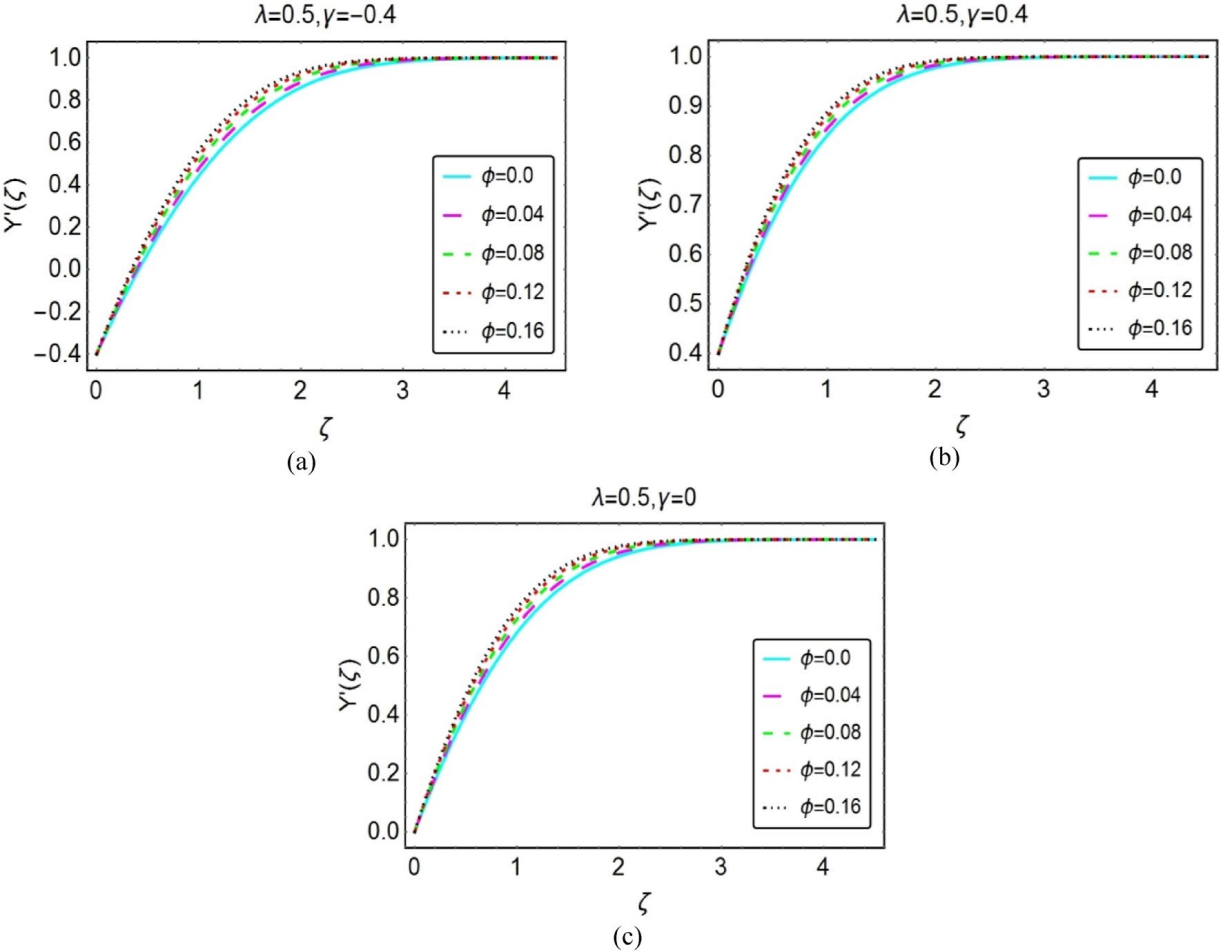
Impacts of $\phi$ on $Y{^{\prime}}(\zeta )$ for (**a**) opposing (**b**) assisting and (**c**) static case.

**Figure 3:**
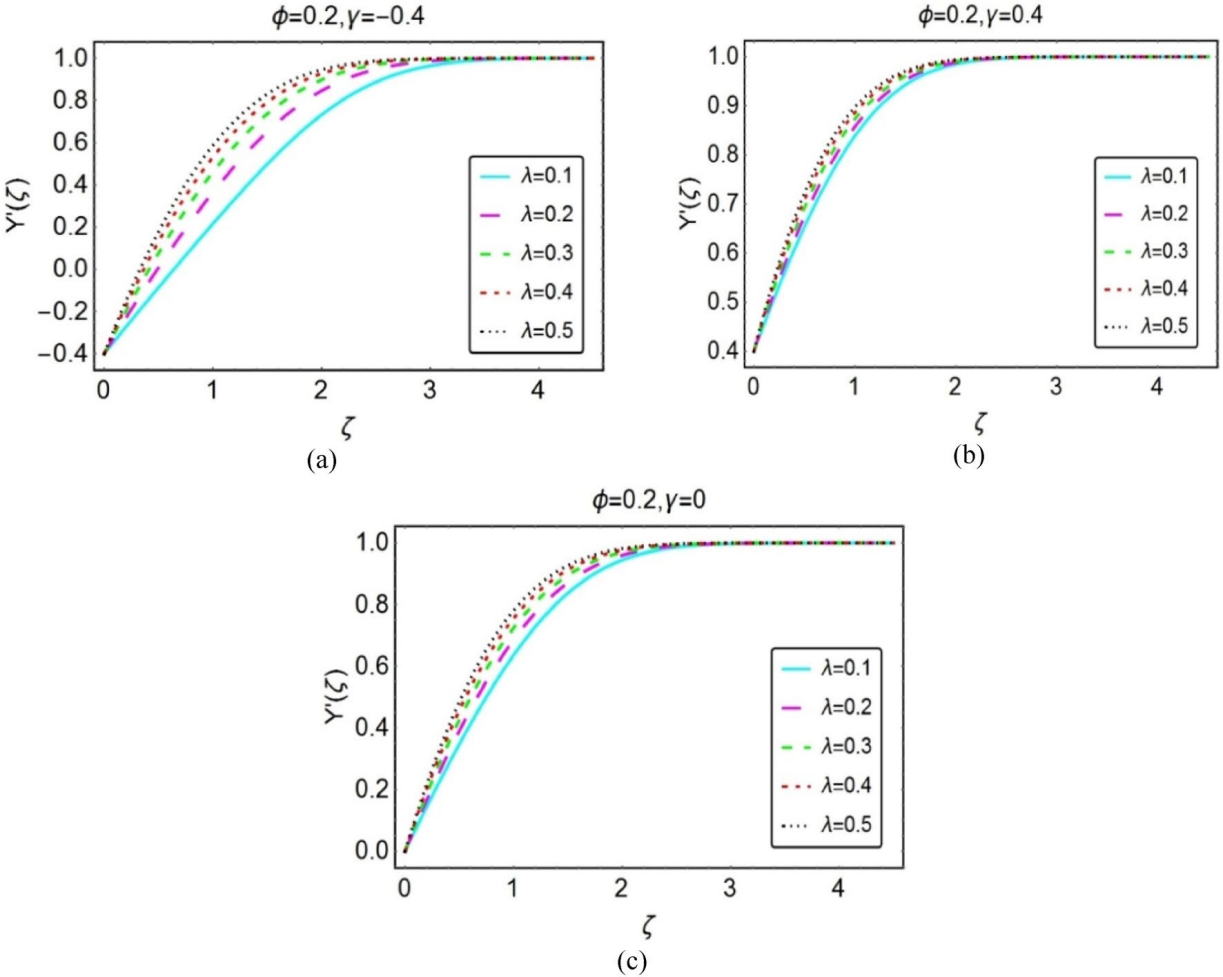
Impacts of $\lambda$ on $Y{^{\prime}}(\zeta )$ for (**a**) opposing (**b**) assisting and (**c**) static case.

Figures 2 and 3 organized to inspect the behaviour of nanofluid velocity $Y{^{\prime}}(\zeta )$ over opposing, assisting and stationary wedge cases, respectively. These results furnished for varying $\phi$ and $\lambda$. The results revealed that the nanofluid velocity drops for both $\phi$ and $\lambda$. However, rapid decays inspected for opposing flow situation. Physically, when fluid and wedge move in reciprocal direction, the frictional force becomes dominant in the fluid layer adjacent to the wedge surface. As a consequent, the velocity $Y{^{\prime}}(\zeta )$ decays; whereas; for assisting flow situation, these variations are quite inconsequential. These results highlighted in Figs. 2 and 3, respectively.

#### The temperature field

**Figure 4:**
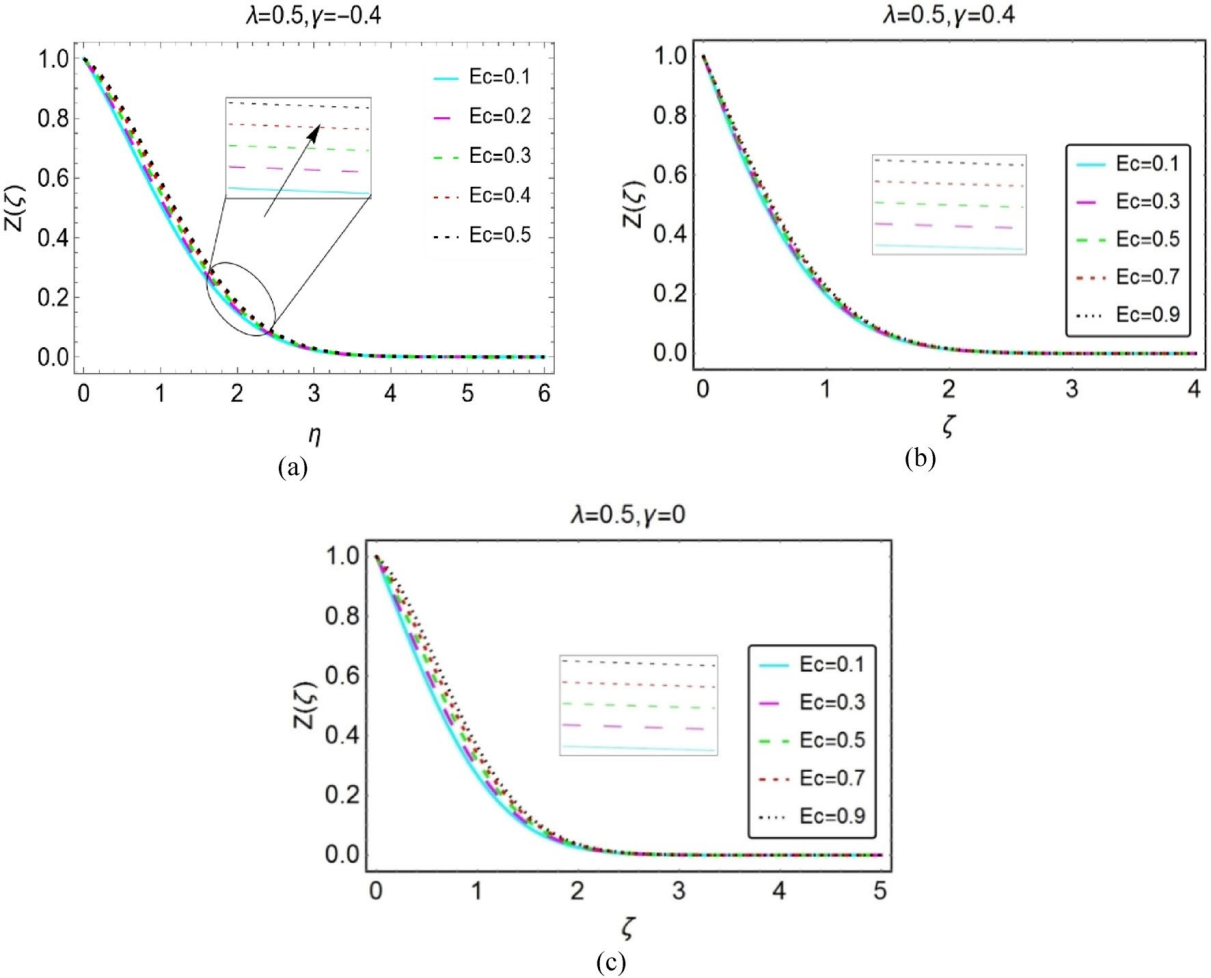
Impacts of Eckert number on $Z(\zeta )$ for (**a**) opposing (**b**) assisting and (**c**) static case.

**Figure 5:**
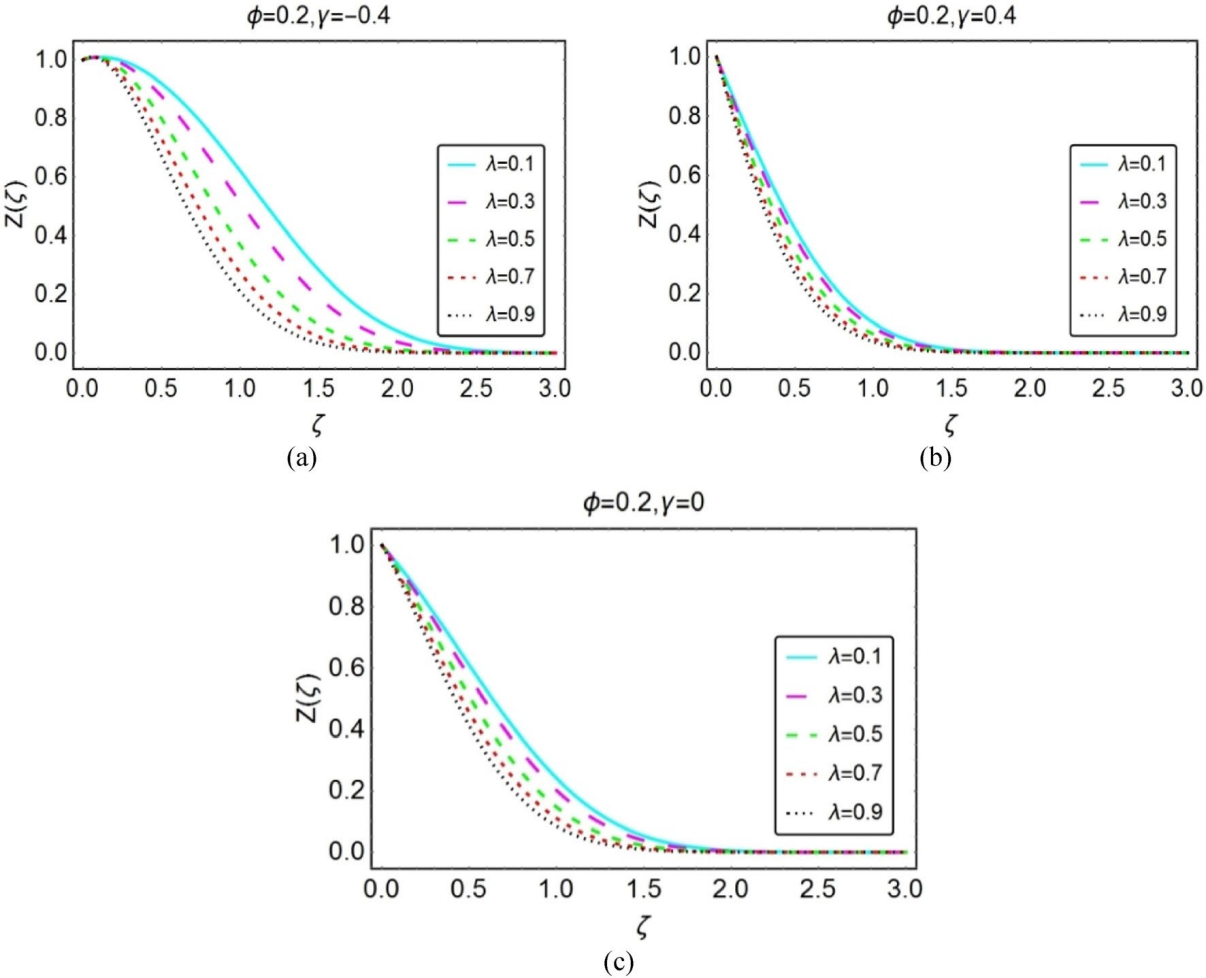
Impacts of $\lambda$ on $Z(\zeta )$ for (**a**) opposing (**b**) assisting and (**c**) static case.

**Figure 6:**
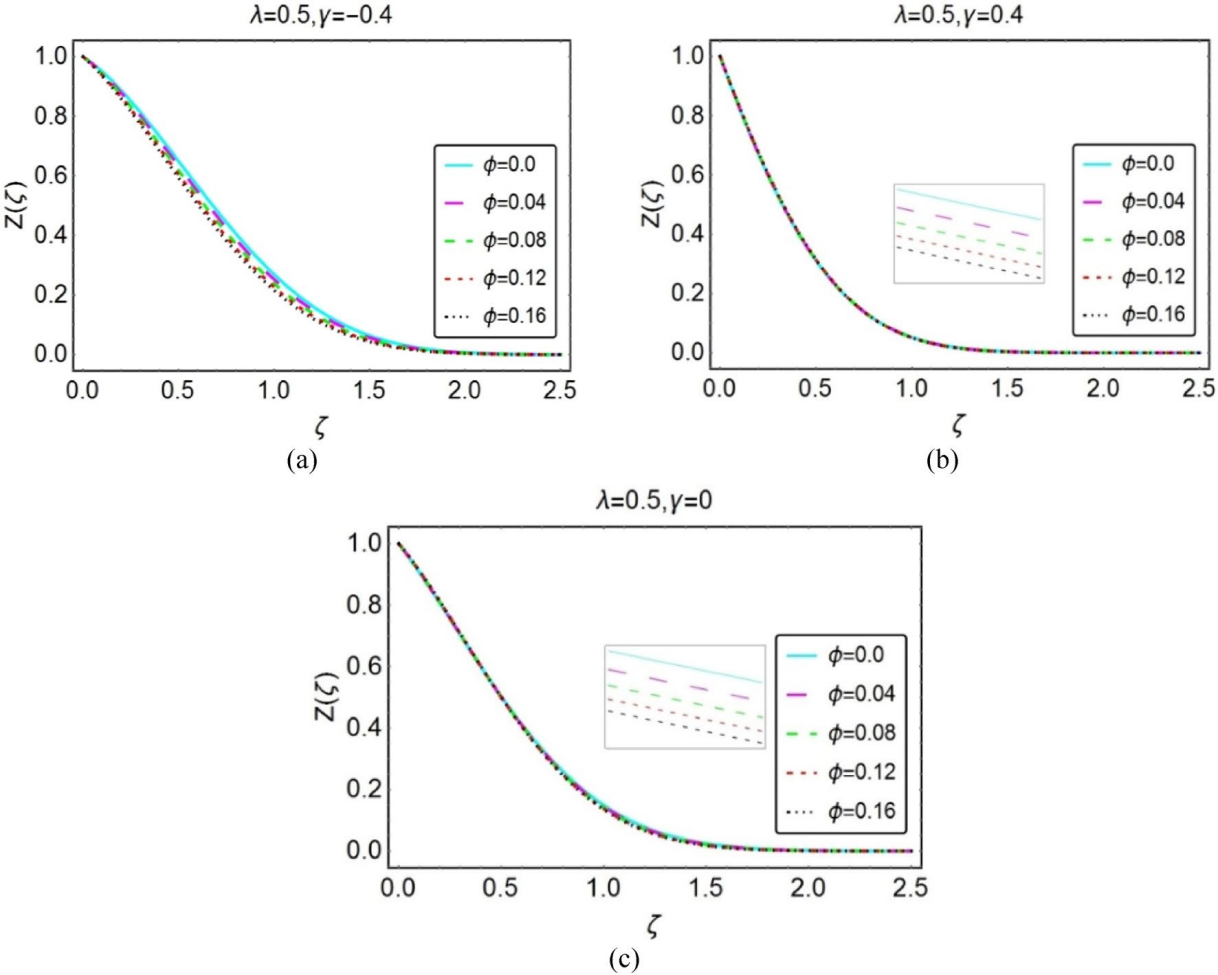
Impacts of $\varnothing$ on $Y(\zeta )$ for (**a**) opposing (**b**) assisting and (**c**) static case.

**Figure 7:**
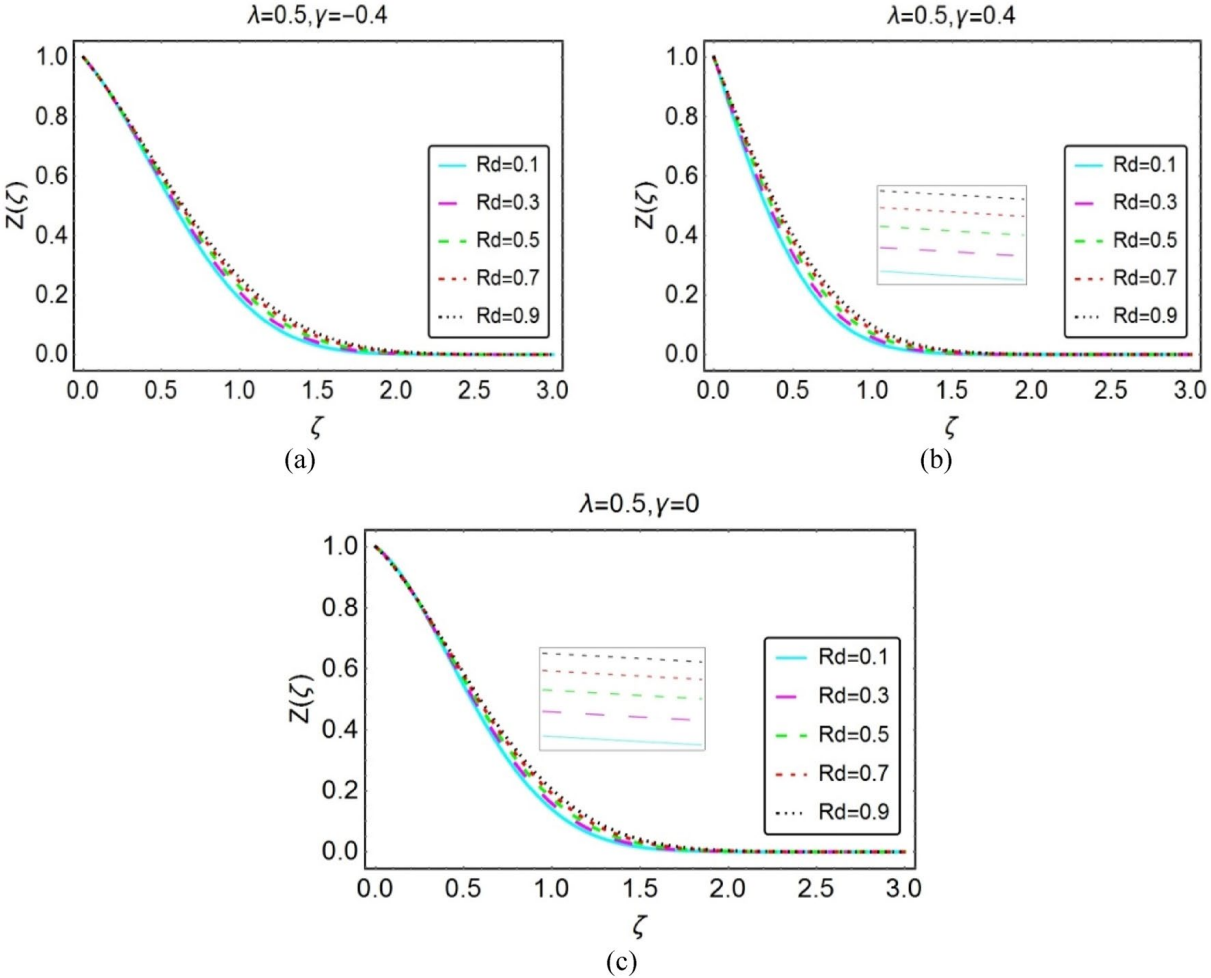
Impacts of $Rd$ on $Z(\zeta )$ for (**a**) opposing (**b**) assisting and (**c**) static case.

This section is organized to analyze the temperature behaviour by varying the flow quantities Eckert number, $\lambda$, $\phi$ and thermal radiation number (Rd). For this, Figs. 4, 5, 6, and 7 under varying parameters effects.

Figure 4a–c decorated to investigate the temperature behaviour against multiple values of Ec for opposing ($\lambda <0$), assisting ($\lambda >0$) and stationary wedge ($\lambda =0$), respectively. It is noticed that Eckert number potentially contributed in the heat transfer of the nanofluid. The significant rise in the temperature is observed for all the cases. Physically, induction of viscous dissipation in the energy equation, improves internal energy of the fluid; consequently, the fluid temperature upshots. The temperature at ambient position of wedge becomes almost inconsequential and asymptotic behaviour is observed.

Figures 5 and 6 highlight the temperature $Z(\zeta )$ for numerous values of $\lambda$ and volume fraction of the nanoparticles $\phi$, respectively. It is noted that these parameters oppose the nanofluids temperature and optimum decrement is noticed for opposing flow cases. Physically, the fluid velocity reduces when wedge and fluid accelerate in opposite direction. As a result, the fluid motion decays and colloisions between the particles declines which lead to rapid declines in the temperature.

The potential contribution of thermal radiation in the temperature field of the nanofluid is decorated in Fig. 7 for three cases (opposing, assisting and stationary wedge). The results revealed that the temperature upsurges in the presence of thermal radiations. Physically, thermal radiations induct energy in the fluid due to which this energy transfers from one to other particles and consequently overall the nanofluid temperature rises. These results highlighted in Fig. 7a–c, respectively.

### Validation of the study

As, the conventional fluid model can be obtained from the nanofluid model by setting $\phi =0.0$. The current study is now validated with previously reported studies by restricting our model for certain flow parameters ($\phi =0,, \lambda =\frac{2m}{m+1}, Rd=0, \gamma =0$). The comparison revealed that the results obtained in the study are in excellent agreement with existing literaure^37,38^ (Table 2).

**Table 2:** Validation of the study for $F{^{\prime}}{^{\prime}}(0)$ under certain conditions on the flow parameters.

$$\phi =0,, \lambda =\frac{2m}{m+1}, Rd=0, \gamma =0$$			
$m$	Current results	Watanbe^37^	Ahmed et al.^38^
$0.0000$	$0.46959$	$0.46960$	$0.46959$
$0.0141$	$0.504614$	–	$0.504614$
$0.0435$	$0.568977$	$0.56898$	$0.568977$
$0.0909$	$0.654978$	$0.65498$	$0.654978$
$0.1429$	$0.731998$	$0.73200$	$0.731998$
$0.2000$	$0.802125$	$0.80213$	$0.802125$
$0.3333$	$0.927653$	$0.92765$	$0.927653$
$0.5000$	$1.038903$	$1.03890$	$1.038903$

## Concluding remarks

The study of nanofluid is reported over a wedge for assisting/opposing flow situations. The flow problem properly modeled by engaging similarity equations and nanofluids effective correlations. The resultant model is treated numerically and furnished the results for assisting/opposing flow. The study revealed that:

- The velocity field rises by increasing the values of $\lambda$.
- The temperature field of the nanofluid significantly upshots for more dissipative fluid and maximum increment is observed for opposing flow case.
- In the presence of thermal radiations, temperature of the nanofluid enhances for both assisting and opposing cases.
- The rapid drops in the temperature field are noticed against the parameter $\lambda$ for considered cases.
- A comparative analysis under certain restrictions is provided with previously published and found an excellent agreement.

## Data Availability

The authors declared no additional data for this manuscript.

## List of symbols

[u^*^, v^*^]: Velocity components
$$\check{T}$$: Temperature
$u_{w}^{*} \left( x \right)$: Velocity at the surface
$U^{*} \left( x \right)$: Main stream velocity
$${\check{T}}_{\infty }$$: Ambient temperature
$${\Psi }^{*}$$: Stream function
$$\eta$$: Dimensionless variable
$$Z\left( \eta \right)$$: Dimensionless temperature
$$\rho_{nf}$$: Effective density
$\left[ {\rho_{s} , \rho_{f} } \right]$: Density of particles and host fluid
$$\mu_{nf}$$: Dynamic viscosity
$\mu_{f}$: Dynamic viscosity of the host fluid
$\left( {\rho c_{p} } \right)_{nf}$: Heat capacity of the nanofluid
$\left( {\rho c_{p} } \right)_{s}$: Heat capacity of the particles
$\left( {\rho c_{p} } \right)_{f}$: Heat capacity of the fluid
$k_{nf}$: Nanofluids thermal conductance
$k_{s}$: Particles thermal conductance
$k_{f}$: Host fluid thermal conductance
$u_{B}$: Brownian velocity of nanoparticles
$$k_{b}$$: Boltzmann constant
$$d_{f}$$: Molecular diameter
$$Y^{\prime}\left( \eta \right)$$: Dimensionless velocity
$$Z\left( \eta \right)$$: Dimensionless temperature
Pr: Prandtl number
Ec: Eckert number
$$\gamma$$: Wedge parameter
Rd: Thermal radiation number
